# Oral-facial-digital syndrome type VI: is *C5orf42* really the major gene?

**DOI:** 10.1007/s00439-014-1508-3

**Published:** 2014-11-19

**Authors:** Marta Romani, Francesca Mancini, Alessia Micalizzi, Andrea Poretti, Elide Miccinilli, Patrizia Accorsi, Emanuela Avola, Enrico Bertini, Renato Borgatti, Romina Romaniello, Serdar Ceylaner, Giangennaro Coppola, Stefano D’Arrigo, Lucio Giordano, Andreas R. Janecke, Mario Lituania, Kathrin Ludwig, Loreto Martorell, Tommaso Mazza, Sylvie Odent, Lorenzo Pinelli, Pilar Poo, Margherita Santucci, Sabrina Signorini, Alessandro Simonati, Ronen Spiegel, Franco Stanzial, Maja Steinlin, Brahim Tabarki, Nicole I. Wolf, Federica Zibordi, Eugen Boltshauser, Enza Maria Valente

**Affiliations:** 1 Lab. Mendel, IRCCS Casa Sollievo della Sofferenza, Viale Regina Margherita 261, 00198 Rome, Italy; 2 Department of Biological and Environmental Science, University of Messina, Messina, Italy; 3 Section of Pediatric Neuroradiology, Division of Pediatric Radiology, The Johns Hopkins School of Medicine, Baltimore, MD USA; 4 Pediatric Neuropsychiatric Division, Spedali Civili, Brescia, Italy; 5 Unit of Pediatrics and Medical Genetics, I.R.C.C.S. Associazione Oasi Maria Santissima, Troina, Italy; 6 Unit of Neuromuscular and Neurodegenerative Disorders, Laboratory of Molecular Medicine, Bambino Gesù Children’s Research Hospital, Rome, Italy; 7 Neuropsychiatry and Neurorehabilitation Unit, Scientific Institute, IRCCS Eugenio Medea, Bosisio Parini, Lecco Italy; 8 Intergen Genetic Diagnosis, Research and Education Center, Ankara, Turkey; 9 Section of Neuroscience, Department of Medicine and Surgery, University of Salerno, Salerno, Italy; 10 Developmental Neurology Division, Fondazione IRCCS Istituto Neurologico C. Besta, Milan, Italy; 11 Department of Pediatrics I and Division of Human Genetics, Innsbruck Medical University, Innsbruck, Austria; 12 Preconceptional and Prenatal Physiopathology, Galliera Hospital, Genoa, Italy; 13 Surgical Pathology and Cytopathology Unit, Department of Medicine (DIMED), University of Padova, Padua, Italy; 14 Department of Molecular Genetics, Hospital Sant Joan de Déu, Barcelona, Spain; 15 Service de Génétique Médicale, CHU Hôpital Sud, Rennes, France; 16 Department of Neuroradiology, Spedali Civili, Brescia, Italy; 17 Department of Neurology, Hospital Sant Joan de Déu, Barcelona, Spain; 18 Pediatric Neuropsychiatry Unit, IRCCS Istituto di Scienze Neurologiche, Bologna, Italy; 19 Unit of Child Neurology and Psychiatry, Centre of Child Neuro-ophthalmology, C. Mondino National Neurological Institute, Pavia, Italy; 20 Department of Neurological Sciences and Movement-Neurology (Child Neurology), University of Verona, Verona, Italy; 21 Emek Medical Center, Genetic Institute, Afula, Israel; 22 Department of Pediatrics, Genetic Counselling Service, Regional Hospital of Bolzano, Bolzano, Italy; 23 Department of Pediatric Neurology, University Children’s Hospital, Bern, Switzerland; 24 Division of Pediatric Neurology, Prince Sultan Military Medical City, Riyadh, Saudi Arabia; 25 Department of Child Neurology, VU University Medical Center and Neuroscience Campus Amsterdam, Amsterdam, The Netherlands; 26 Department of Child Neurology, Fondazione IRCCS Istituto Neurologico “Carlo Besta”, Milan, Italy; 27 Department of Pediatric Neurology, University Children’s Hospital, Zurich, Switzerland; 28 Neurogenetics Unit, CSS-Mendel Institute, Viale Regina Margherita 261, 00198 Rome, Italy

## Abstract

Oral-facial-digital type VI syndrome (OFDVI) is a rare phenotype of Joubert syndrome (JS). Recently, *C5orf42* was suggested as the major OFDVI gene, being mutated in 9 of 11 families (82 %). We sequenced *C5orf42* in 313 JS probands and identified mutations in 28 (8.9 %), most with a phenotype of pure JS. Only 2 out of 17 OFDVI patients (11.7 %) were mutated. A comparison of mutated vs. non-mutated OFDVI patients showed that preaxial and mesoaxial polydactyly, hypothalamic hamartoma and other congenital defects may predict *C5orf42* mutations, while tongue hamartomas are more common in negative patients.

**Electronic supplementary material:**

The online version of this article (doi:10.1007/s00439-014-1508-3) contains supplementary material, which is available to authorized users.

Oral-facial-digital type VI syndrome (OFDVI) is a rare phenotype in the spectrum of Joubert syndrome (JS) and is defined by the presence of the “molar tooth sign” (MTS) with at least one of these findings: (1) tongue hamartoma and/or additional lingual frenula and/or upper lip notch; (2) mesoaxial polydactyly; (3) hypothalamic hamartoma. Other oral-facial (e.g. cleft lip and palate) or digital (e.g. postaxial and preaxial polydactyly) abnormalities can also be present (Poretti et al. 2012).

Mutations in *TMEM216* and in *OFD1* have been reported in few OFDVI patients (Coene et al. 2009; Darmency-Stamboul et al. 2013; Valente et al. 2010). A recent study identified mutations in the *C5orf42* gene in nine of 11 OFDVI (82 %) families (including four living children and eight fetuses), suggesting that *C5orf42* could represent the major causative gene for OFDVI (Lopez et al. 2014).

As part of a ciliopathy research project, we sequenced *C5orf42* in 313 JS probands, and identified pathogenic mutations in 28 (8.9 %) (Fig. 1). Only two out of 17 OFDVI probands in our cohort (11.7 %) carried *C5orf42* mutations, while one was mutated in *OFD1*. No mutations were detected in the remaining 14 (82.3 %) OFDVI patients in all tested genes (see Supplementary material online for methods, characterization of mutations and clinical features of mutated OFDVI patients).

**Fig. 1:**
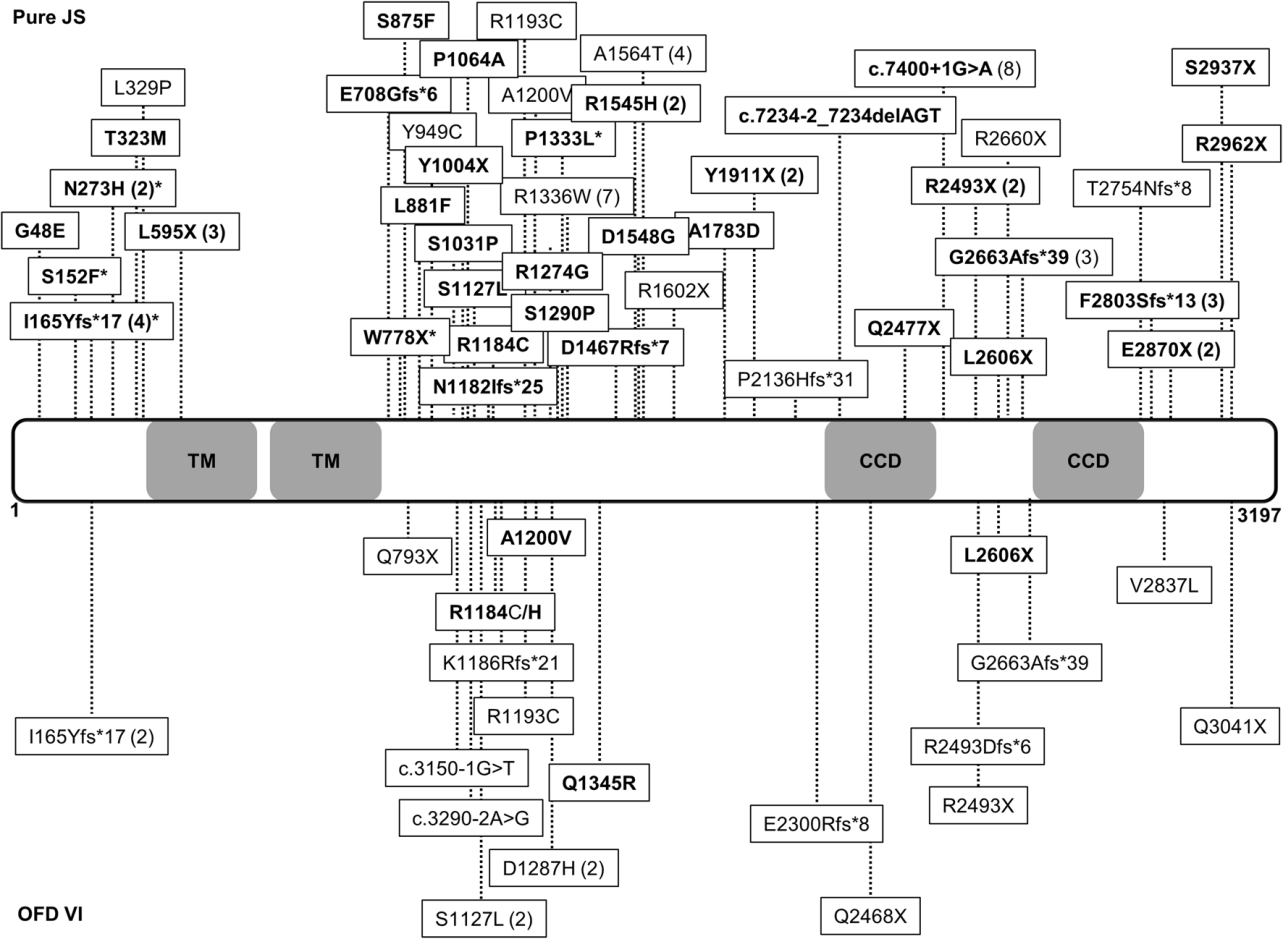
Schematic representation of C5orf42 protein structure and distribution of all reported mutations. The two predicted transmembrane domains (TM, amino acids 592–612 and 631–651) and the two predicted coiled coil domains (CCD, amino acids 2,457–2,487 and 2,691–2,724) are shown. Mutations found in patients with pure Joubert syndrome and with OFDVI are presented in the *upper* and *lower parts* of the figure, respectively. Mutations identified in the present study are in *bold*. In *brackets* are the numbers of patients in whom each mutation has been identified. *Asterisk* indicates clinical data not available

To explain the striking discrepancy between our findings and those reported by Lopez et al., we compared clinical features in *C5orf42* mutated (*n* = 14) vs. non-mutated (*n* = 17) OFDVI patients (Table 1). Preaxial and mesoaxial polydactyly, hypothalamic hamartomas and other congenital abnormalities were significantly more frequent in the mutated group, while tongue hamartomas or multiple lingual frenula occurred more commonly in non-mutated patients. Other oral-facial features, postaxial polydactyly and other brain abnormalities were equally represented in both groups. Despite the limited number of patients, these findings suggest that the current diagnostic criteria for OFDVI include two main phenotypic groups, one with preaxial and/or mesoaxial polydactyly and frequent additional congenital anomalies (for which *C5orf42* is the major causative gene), and another with less severe presentation and prevalent oral-facial involvement, which genetic causes still remain to be identified.

**Table 1:** Comparison of clinical features in *C5orf42* mutated vs. non-mutated OFDVI patients

	Mutated	Non-mutated	*p*
Any oral-facial feature	7/12 (58 %)	17/17 (100 %)	0.006
Tongue hamartomas/multiple lingual frenula^a^	6/12 (50 %)	17/17 (100 %)	0.002
Other oral-facial features^b^	4/12 (33 %)	5/17 (29 %)	n.s.
Any polydactyly	14/14 (100 %)	13/17 (76 %)	n.s.
Mesoaxial polydactyly^a^	7/14 (50 %)	1/17 (6 %)	0.01
Preaxial polydactyly	14/14 (100 %)	5/17 (29 %)	0.0001
Postaxial polydactyly	9/14 (64 %)	10/17 (59 %)	n.s.
Any CNS abnormality besides MTS	8/14 (57 %)	4/17 (24 %)	n.s.
Hypothalamic hamartoma^a^	6/14 (43 %)	1/17 (6 %)	0.03
Occipital encephalocele	2/14 (14 %)	1/17 (6 %)	n.s.
Other CNS abnormalities^c^	4/14 (29 %)	2/17 (12 %)	n.s.
Retinal/renal/hepatic involvement	0/14	4/17 (24 %)	n.s.
Retinopathy (only living patients)	0/2	3^e^/17 (18 %)	n.s.
Nephronophthisis (only living patients)	0/2	2^e^/17 (12 %)	n.s.
Cystic dysplastic kidneys	0/14	0/17	n.s.
Congenital liver fibrosis	0/14	0/17	n.s.
Other congenital abnormalities outside the CNS^d^	8/14 (57 %)	1/17 (6 %)	0.004

*C5orf42* mutated patients include the 12 patients from 9 families reported by Lopez et al. (2014) and the two patients from the present paper; *C5orf42* non-mutated patients (*n* = 17) are all from the present cohort, and include one patient mutated in *OFD1* (see text) and 16 patients from 14 families. Statistical comparisons were made by Fisher’s exact test

^a^Sufficient for diagnosis of OFDVI in association with the MTS

^b^Cleft lip and/or palate, tooth abnormalities, lobulated tongue, short frenula

^c^Porencephaly, nodular heterotopia, polymicrogyria, corpus callosum abnormalities, hydrocephalus, arhinencephaly

^d^Abnormal ribs or long bones, cubitus valgus, heart or aortic defects, uterus septation, common mesentery, coloboma, microphthalmia, Hirschsprung disease, scoliosis

^e^Includes two siblings

Twenty-seven *C5orf42* mutated patients (from 23 families) in our study had pure JS (with retinopathy in one), while clinical data were unavailable in three. Considering all reported *C5orf42* mutated patients (*n* = 58), over two-thirds showed a pure JS phenotype while only 24 % has OFDVI (Supplementary Table 1). Kidney or liver involvement was never noted, while polydactyly (mainly preaxial) was present in nearly half of mutated patients regardless of the phenotype. These findings delineate a specific *C5orf42*-related phenotype, and suggest a major role for this gene in limb development.

Overall, the identification of mutations in 28 of 313 JS probands makes *C5orf42* a major contributor to the pathogenesis of this ciliopathy. How mutations in the same gene may cause pure JS or a much more severe oral-facial-digital syndrome remains an open question. Genotype–phenotype correlations seem to fail, since truncating and missense mutations affecting the entire length of the protein are detected in patients with either pure or OFDVI presentations (Fig. 1). As suggested for other ciliopathies, it is conceivable that additional, yet unidentified variants in distinct genes may act as genetic modifiers able to influence the penetrance and expression of oral-facial and digital features in patients bearing *C5orf42* mutations.

## Electronic supplementary material

Below is the link to the electronic supplementary material.

Supplementary material 1 (DOCX 39 kb)
