# Supplementary material for: Oral-facial-digital syndrome type VI: is C5orf42 really the major gene?
Source: Hum Genet. 2014 Nov 19;134(1):123–6. doi: 10.1007/s00439-014-1508-3 (PMC4282684; doi:10.1007/s00439-014-1508-3)
Supplement: Supplementary file 1 — Supplementary material 1 (DOCX 39 kb) [file 439_2014_1508_MOESM1_ESM.docx]

# Journal: Human Genetics

# Oral-facial-digital syndrome type VI: is *C5orf42* really the major gene?

Marta Romani, PhD^1^ Francesca Mancini, MD^1^ Alessia Micalizzi, BSc^1,2^ Andrea Poretti, MD^3^ Elide Miccinilli, BSc ^1^ Patrizia Accorsi, MD^4^ Emanuela Avola, MD^5^ Enrico Bertini, MD^6^ Renato Borgatti, MD^7^ Romina Romaniello, MD^7^ Serdar Ceylaner, MD^8^ Giangennaro Coppola, MD^9^ Stefano D’Arrigo, MD^10^ Lucio Giordano, MD^4^ Andreas R. Janecke, MD^11^ Mario Lituania, MD^12^ Kathrin Ludwig, MD^13^ Loreto Martorell,^14^ Tommaso Mazza, PhD^1^ Sylvie Odent, MD^15^ Lorenzo Pinelli, MD^16^ Pilar Poo, MD,^17^ Margherita Santucci, MD^18^ Sabrina Signorini, MD, PhD^19^ Alessandro Simonati, MD^20^ Ronen Spiegel, MD^21^ Franco Stanzial, MD^22^ Maja Steinlin, MD^23^ Brahim Tabarki, MD^24^ Nicole I. Wolf,^25^ Federica Zibordi, MD^26^ Eugen Boltshauser, MD^27^ Enza Maria Valente MD, PhD^1,9^

^1^IRCCS Casa Sollievo della Sofferenza, Mendel Laboratory, San Giovanni Rotondo, Italy; ^2^ Department of Biological and Environmental Science, University of Messina, Italy; ^3^Section of Pediatric Neuroradiology, Division of Pediatric Radiology, The Johns Hopkins School of Medicine, Baltimore, MD, USA ; ^4^Pediatric Neuropsychiatric Division, Spedali Civili, Brescia, Italy; ^5^ Unit of Pediatrics and Medical Genetics, I.R.C.C.S. Associazione Oasi Maria Santissima, Troina, Italy; ^6^Unit of Neuromuscular and Neurodegenerative Disorders, Laboratory of Molecular Medicine, Bambino Gesù Children’s Research Hospital, Rome, Italy; ^7^Neuropsychiatry and Neurorehabilitation Unit, Scientiﬁc Institute, IRCCS Eugenio Medea, Bosisio Parini, Lecco; ^8^Intergen Genetic Diagnosis, Research and Education Center, Ankara, Turkey; ^9^Section of Neuroscience, Department of Medicine and Surgery, University of Salerno, Salerno, Italy; ^10^Developmental Neurology Division, Fondazione IRCCS Istituto Neurologico C. Besta, Milano, Italy; ^11^Department of Pediatrics I and Division of Human Genetics, Innsbruck Medical University, Innsbruck, Austria; ^12^Preconceptional and Prenatal Physiopathology, Galliera Hospital; ^13^Surgical Pathology and Cytopathology Unit, Department of Medicine (DIMED), University of Padova, Padova, Italy; ^14^Department of Molecular Genetics, Hospital Sant Joan de Déu, Barcelona, Spain ; ^15^Service de Génétique Médicale, CHU Hôpital Sud, Rennes, France; ^16^Department of Neuroradiology, Spedali Civili, Brescia, Italy; ^17^Department of Neurology, Hospital Sant Joan de Déu, Barcelona, Spain ; ^18^Pediatric Neuropsychiatry Unit, IRCCS Istituto di Scienze Neurologiche, Bologna, Italy; ^19^Centre of Child Neuro-ophthalmology, Unit of Child Neurology and Psychiatry, C. Mondino National Neurological Institute, Pavia; ^20^Department of Neurological Sciences and Movement-Neurology (Child Neurology), University of Verona, Verona, Italy; ^21^Genetic Institute, Emek Medical Center, Afula, Israel; ^22^Department of Pediatrics, Genetic Counselling Service, Regional Hospital of Bolzano, Bolzano, Italy; ^23^ Department of Pediatric Neurology, University Children’s Hospital, Berne, Switzerland ; ^24^Division of Pediatric Neurology, Prince Sultan Military Medical City, Riyadh, Saudi Arabia; ^25^Department of Child Neurology, VU University Medical Center and Neuroscience Campus Amsterdam, Amsterdam, The Netherlands; ^26^Department of Child Neurology, Fondazione IRCCS Istituto Neurologico “Carlo Besta,” Milan, Italy; ^27^Department of Pediatric Neurology, University Children’s Hospital, Zurich, Switzerland.

**Corresponding author:**

Prof. Enza Maria Valente, MD, PhD

Neurogenetics Unit

CSS-Mendel Institute

Viale Regina Margherita 261

00198 Rome, Italy

Ph: +39 06 4416 0537

Fax: +39 06 4416 0548

Email: e.valente@css-mendel.it

## Supplementary material

## Methods

Patients’ cohort included a total of 313 probands representative of the whole clinical spectrum of JS, recruited by the unique neuroimaging criterion of the molar tooth sign (MTS). Among them, 17 living patients matched the diagnostic criteria for OFDVI (Poretti et al. 2012). For each patient, a standardized clinical questionnaire filled by the referring clinician allowed to obtain detailed information on the phenotypic spectrum and the extent of organ involvement. Written informed consent was obtained from all families, and the study was approved by the local ethics committee.

All patients underwent simultaneous target sequencing of 50 ciliopathy genes (see Supplementary Table 2), including the *C5orf42* gene and other 21 genes causative of Joubert syndrome), on a Solid 5500xL platform (Life Technologies). Sensitivity of the technique was assessed by sequencing 54 patients already known to carry point mutations or very small insertions/deletions in several JS causative genes: each previously identified mutation (either in the compound heterozygous or homozygous state) could be confirmed by target sequencing on the Solid platform, demonstrating a very high sensitivity of the adopted protocol.

To amplify *C5orf42*, probes have been designed to cover each of the 52 exons of the longest isoform of the gene (NM_023073, encoding a 3197 amino acid protein), with splice-site junctions and at least 30 bp of flanking introns. Due to the very high coverage obtained (mean depth 400X), we could verify that each base pair of the *C5orf42* coding sequence was covered at least 20X in every patient. Nevertheless, it must be said that next generation sequencing techniques might fail to detect certain types of mutations (such as larger insertions or deletions), and therefore we cannot be sure that our *C5orf42* mutation frequency could be slightly underestimated.

All identified mutations in *C5orf42*  were validated using bidirectional Sanger sequencing. Confirmed mutations were searched against public databases dbSNP ver.141 (<http://www.ncbi.nlm.nih.gov/SNP/>) and Exome Variant Server (<http://evs.gs.washington.edu/EVS/>), and their potential pathogenicity was predicted using prediction software PolyPhen-2 ver.2.2.2 (<http://genetics.bwh.harvard.edu/pph2/>) and SIFT (<http://sift.jcvi.org/>). Nomenclature was assigned according to the Human Genome Variant Society (<http://www.hgvs.org/mutnomen/>).

## Characterization of C5orf42 mutations

In this work, we identified 37 distinct mutations in the *C5orf42* gene (of which 30 novel), including 19 missense, 10 nonsense, 6 frameshift and 2 splice-site mutations (Figure 1). Thirty-three mutations were not found in public databases dbSNP ver.141 and Exome Variant Server, while four were present in dbSNP/EVS with extremely low (0.0077 to 0.021%) or no reported minor allele frequency, and never in the homozygous state. All novel missense mutations were predicted as damaging or not tolerated by both prediction web tools.

## Clinical features of *C5orf42* mutated OFDVI probands

### Patient 1

Patient NG3674 is a 4 years-old boy born from non-consanguineous parents, compound heterozygous for *C5orf42* missense mutation c.C3599T; p.A1200V and nonsense mutation c.T7817A; p.L2606X. Fetal ultrasound at 26 gestational weeks showed an enlarged fourth ventricle. His neonatal period was characterized by breathing abnormalities, hypotonia and nystagmus. Clinical examination showed mild intellectual impairment and mesoaxial polydactyly of hands and preaxial polydactyly of feet, in the absence of any clear oral features. Visual evoked potentials were reduced bilaterally. A brain MRI showed the MTS.

### Patient 2

Patient NG1610, a 12 years-old boy born from unrelated parents, was compound heterozygous for the two *C5orf42* missense mutations c.G3551A; p.R1184H and c.A4034G; p.Q1345R. Pregnancy and delivery were unremarkable. He had a complex phenotype characterized by severe developmental delay, ataxia, ocular motor apraxia, preaxial polydactyly of hands and feet, tongue hamartomas and multiple lingual frenula. He was diagnosed with Hirschsprung disease, while renal, hepatic and retinal functions were normal. Brain MRI at age 2 years showed the MTS associated with hypothalamic hamartoma, thin corpus callosum and bilateral polymicrogyria. This patient had been previously reported in Poretti et al. 2012 (patient 11).

## Supplementary References

Alazami AM, Alshammari MJ, Salih MA, Alzahrani F, Hijazi H, Seidahmed MZ, Abu Safieh L, Aldosary M, Khan AO, Alkuraya FS (2012) Molecular characterization of Joubert syndrome in Saudi Arabia. Hum Mutat 33: 1423-1428. doi: 10.1002/humu.22134

Ohba C, Osaka H, Iai M, Yamashita S, Suzuki Y, Aida N, Shimozawa N, Takamura A, Doi H, Tomita-Katsumoto A, Nishiyama K, Tsurusaki Y, Nakashima M, Miyake N, Eto Y, Tanaka F, Matsumoto N, Saitsu H (2013) Diagnostic utility of whole exome sequencing in patients showing cerebellar and/or vermis atrophy in childhood. Neurogenetics 14: 225-232. doi: 10.1007/s10048-013-0375-8

Poretti A, Brehmer U, Scheer I, Bernet V, Boltshauser E (2008) Prenatal and neonatal MR imaging findings in oral-facial-digital syndrome type VI. AJNR Am J Neuroradiol 29: 1090-1091. doi: 10.3174/ajnr.A1038

Romani M, Micalizzi A, Valente EM (2013) Joubert syndrome: congenital cerebellar ataxia with the molar tooth. Lancet Neurol 12: 894-905. doi: 10.1016/S1474-4422(13)70136-4

Shaheen R, Faqeih E, Alshammari MJ, Swaid A, Al-Gazali L, Mardawi E, Ansari S, Sogaty S, Seidahmed MZ, Almotairi MI, Farra C, Kurdi W, Al-Rasheed S, Alkuraya FS (2013) Genomic analysis of Meckel-Gruber syndrome in Arabs reveals marked genetic heterogeneity and novel candidate genes. Eur J Hum Genet 21: 762-768. doi: 10.1038/ejhg.2012.254

Srour M, Hamdan FF, Schwartzentruber JA, Patry L, Ospina LH, Shevell MI, Desilets V, Dobrzeniecka S, Mathonnet G, Lemyre E, Massicotte C, Labuda D, Amrom D, Andermann E, Sebire G, Maranda B, Rouleau GA, Majewski J, Michaud JL (2012a) Mutations in TMEM231 cause Joubert syndrome in French Canadians. J Med Genet 49: 636-641. doi: 10.1136/jmedgenet-2012-101132

Srour M, Schwartzentruber J, Hamdan FF, Ospina LH, Patry L, Labuda D, Massicotte C, Dobrzeniecka S, Capo-Chichi JM, Papillon-Cavanagh S, Samuels ME, Boycott KM, Shevell MI, Laframboise R, Desilets V, Maranda B, Rouleau GA, Majewski J, Michaud JL (2012b) Mutations in C5ORF42 cause Joubert syndrome in the French Canadian population. Am J Hum Genet 90: 693-700. doi: 10.1016/j.ajhg.2012.02.011

## Supplementary Table 1 – Prevalence of clinical features in *C5orf42* mutated patients reported to date

|  | **Srour 2012b** | **Srour 2012a** | **Alazami 2012** | **Ohba 2013** | **Lopez 2014** | **Shaheen 2013** | **Present study** | **TOTAL (%)** |
| --- | --- | --- | --- | --- | --- | --- | --- | --- |
| **Nr. of patients** (including fetuses with confirmed diagnosis) | 10 | 1 | 3 | 2 | 12 | 1 | 29 | **58** |
| **Clinical phenotype:** |  |  |  |  |  |  |  |  |
| - pure JS | 10 | 1 | 2 | 2 | - | - | 26* (1 fetus) | **41 (70.7%)** |
| - JS with retina | - | - | 1 | - | - | - | 1 | **2 (3.4%)** |
| - JS with kidney | - | - | - | - | - | - | - | **-** |
| - cerebello-oculo-renal | - | - | - | - | - | - | - | **-** |
| - JS with liver | - | - | - | - | - | - | - | **-** |
| - OFDVI | - | - | - | - | 12 (8 fetuses) | - | 2 | **14 (24.1%)** |
| - MKS-like fetuses | - | - | - | - | - | 1 | - | **1 (1.7%)** |
| **Specific clinical features:** |  |  |  |  |  |  |  |  |
| - neurological signs (living pts) | 10 | 1 | 3 | 2 | 4 | - | 28 | **48/48 (100%)** |
| - retinopathy (living pts) | - | - | 1 | - | - | - | 1 | **2/48 (4.2%)** |
| - kidney/liver involvement | - | - | - | - | - | - | 1* | **1/58 (1.7%)** |
| - any oral-facial feature | - | - | - | - | 6 | 1 | 2 | **9/56 (16.1%)** |
| *- tongue hamartomas / multiple lingual frenula^a^* | *-* | *-* | *-* | *-* | *5* | *-* | *1* | *6 (10.7%)* |
| *- other oral-facial features^b^* | *-* | *-* | *-* | *-* | *4* | *1* | *1* | *6 (10.7%)* |
| - any polydactyly | 1 | - | - | - | 12 | - | 15 | **28/58 (48.3%)** |
| *- mesoaxial polydactyly^a^* | *-* | *-* | *-* | *-* | *6* | *-* | *1* | *7 (12.1%)* |
| *- preaxial polydactyly* | *1* | *-* | *-* | *-* | *12* | *-* | *13* | *26 (44.8%)* |
| *- postaxial polydactyly* | *1* | *-* | *-* | *-* | *9* | *-* | *6* | *16 (27.6%)* |
| - any CNS abnormality besides MTS | - | - | 1 | - | 9 | 1 | 4 | **15/58 (25.9%)** |
| *- hypothalamic hamartoma^a^* | *-* | *-* | *-* | *-* | *5* | *-* | *1* | *6 (10.3%)* |
| *- occipital meningoencephalocele* | *-* | *-* | *1* | *-* | *-* | *1* | *2* | *4 (6.9%)* |
| *- other CNS abnormalities^c^* | *-* | *-* | *-* | *-* | *4* | *-* | *2* | *6 (10.3%)* |
| - other congenital abnormalities outside the CNS*^d^* | - | - | - | 1 | 7 | 1 | 4 | **13/58 (22.4%)** |

Legend as in Table 1. Clinical phenotypes as described in Romani et al, 2013. *one patient had an enlarged, non-functioning right kidney from birth

## Supplementary Table 2 - List of sequenced genes

| AHI1 | INPP5E |
| --- | --- |
| ALMS1 | INVS |
| ARL13B | KIF7 |
| ARL6 | MKKS |
| ATXN10 | MKS1 |
| B9D1 | NEK1 |
| B9D2 | NEK8 |
| BBS1 | NPHP1 |
| BBS10 | NPHP3 |
| BBS12 | NPHP4 |
| BBS2 | OFD1 |
| BBS4 | PIK3C2A |
| BBS5 | PTHB1 |
| BBS7 | RPGRIP1L |
| C5ORF42 | SDCCAG8 |
| CC2D2A | TCTN1 |
| CEP290 | TCTN2 |
| CEP41 | TCTN3 |
| EVC | TMEM138 |
| EVC2 | TMEM216 |
| GLI3 | TMEM237 |
| GLIS2 | TMEM67 |
| IFT122 | TRIM32 |
| IFT43 | TTC21B |
| IFT80 | TTC8 |
